# Supplementary material for: Cranial ontogenetic variation in early saurischians and the role of heterochrony in the diversification of predatory dinosaurs
Source: PeerJ. 2016 Jan 18;4:e1589. doi: 10.7717/peerj.1589 (PMC4734445; doi:10.7717/peerj.1589)
Supplement: Supplemental Information 1 — Landmark descriptions, taxon sampling, phylogeny and additional figures and tables. [file peerj-04-1589-s001.docx]

**Supplementary information**

**Cranial ontogenetic variation in early saurischians and the role of heterochrony in the diversification of predatory dinosaurs**

Christian Foth^1,2,3^, Brandon P. Hedrick^4,5^, Martín D. Ezcurra^2,6,7^

^1^ SNBS, Bayerische Staatssammlung für Paläontologie und Geologie, Richard Wagner-Str. 10, D-80333 München

^2^ Department of Earth and Environmental Sciences, Ludwig-Maximilians-Universität, Richard-Wagner-Str. 10, D-80333 München, Germany

^3^ Department of Geosciences, University of Fribourg/Freiburg, Chemin du Musée 6, 1700 Fribourg, Switzerland

^4^ Department of Earth and Environmental Science, University of Pennsylvania, 251 Hayden Hall, 240 S 33rd Street, Philadelphia, PA 19104, USA

^5^ Department of Biology, University of Massachusetts, 321 Morrill Science Center, 611 North Pleasant Street, Amherst, MA 01003, USA

^6^ School of Geography, Earth and Environmental Sciences, University of Birmingham, Edgbaston, Birmingham B15 2TT, UK

^7^ Sección Paleontología de Vertebrados, Museo Argentino de Ciencias Naturales “Bernardino Rivadavia”, Buenos Aires C1405DJR, Argentina

**Content**

1. Landmark description
2. Landmark error after Singleton (2002)
3. Taxon sampling
4. Comments on sliding semi-landmarks
5. Phylogeny
6. Influence of nasal crests on the results
7. Influence of semi-landmarks on the results
8. References

**1. Landmark description**

**Table S1 List of landmarks and semi-landmark description. Semi-landmarks (semi-LMs) are marked with a ‘S’.**

| **LMs** | **Description** |
| --- | --- |
| **1** | Most anterior point of the premaxilla along the tooth row (This point is reconstructed for *Alioramus* and *Zupaysaurus*). |
| **2** | Contact between premaxilla and maxilla along the tooth row. |
| **3** | Contact between the maxilla and jugal along the ventral margin of the skull |
| **4** | Contact between the nasal process of the premaxilla and the anterodorsal process of the nasal along the dorsal margin of the external naris |
| **5** | Most anterior point of the antorbital fossa (in those taxa without an antorbital fossa, the most anterior point of the antorbital fenestra was marked). |
| **6** | Most anterior point of the anterior process of the lacrimal along the dorsal margin of the antorbital fenestra. |
| **7** | Most ventral point of the ventral process of the lacrimal along the posteroventral margin of the antorbital fenestra. |
| **8** | Most posterior point of the jugal process of the maxilla along the ventral margin of the antorbital fenestra. |
| **9** | Most anterior point of the jugal (Depending on the configuration of maxilla, lacrimal and jugal, the landmarks 7, 8 and 9 can be similarly located). |
| **10** | Contact between the ventral process of the lacrimal and the jugal along the anteroventral margin of the orbit. |
| **11** | Contact between the jugal and the postorbital along the posterior margin of the orbit. |
| **12** | Most dorsal point of the anterior process of the postorbital along the posterodorsal margin of the orbit. |
| **13** | Contact between the jugal and the postorbital along the anterior margin of the lateral temporal fenestra. |
| **14** | Ventral contact between postorbital and squamosal along the dorsal margin of the lateral temporal fenestra. |
| **15** | Contact between the descending process of the squamosal with the quadratojugal along the posterior margin of the lateral temporal fenestra (For those taxa, where these bones do not contact to each other, e.g. the juvenile *Coelophysis*, the most anteroventral point of the descending process of the squamosal was marked. |
| **16** | Contact between jugal and quadratojugal along the ventral margin of the lateral temporal fenestra. |
| **17** | Dorsal contact between postorbital and squamosal. |
| **18** | Contact between the descending process of the squamosal with the quadratojugal along the posterior margin of the skull (For those taxa, where these bones do not contact to each other, e.g. the juvenile *Coelophysis*, the most posteroventral point of the descending process of the squamosal was marked. |
| **19** | Posteroventral corner of the quadratojugal. |
| **S20-S22** | Three semi-LMs on the ventral margin of the maxilla from LM 2 to LM 3. |
| **S23-S27** | Five semi-LMs along the narial margin starting and ending in LM 4. |
| **S28-S30** | Three semi-LMs along the anterior margin of the antorbital fenestra from LM 6 to LM 8. |
| **S31** | One semi-LM along the posterior margin of the antorbital fenestra from LM 6 to LM 7. |
| **S32** | One semi-LM along the ventral margin of the orbit from LM 10 to LM 11. |
| **S33-S36** | Four semi-LMs along the anterodorsal margin of the orbit from LM 10 to LM 12. |
| **S37-S39** | Three semi-LMs along the posterodorsal margin of the orbit from LM 12 to LM 11. |
| **S40** | One semi-LM along the anterodorsal margin of the lateral temporal fenestra from LM 13 to LM 14. |
| **S41-S42** | Two semi-LMs along the posterodorsal margin of the lateral temporal fenestra from LM 14 to LM 15. |
| **S43-S44** | Two semi-LMs along the posteroventral margin of the lateral temporal fenestra from LM 15 to LM 16. |
| **S45-S46** | Two semi-LMs along the anteroventral margin of the lateral temporal fenestra from LM 16 to LM 13. |
| **S47-S48** | Two semi-LMs along the ventral margin of the skull from LM 3 to LM 19. |
| **S49-S50** | Two semi-LMs along the posterior margin of the quadratojugal from LM 19 to LM 18. |
| **51** | Most posterior point of the descending process of the maxilla contacting the nasal and/or the lacrimal. |
| **S52-S56** | Five semi-LMs along the anterodorsal margin of the maxilla from LM 2 to LM 51. |
| **S57-S71** | Fifteen semi-LMs along the dorsal margin of the skull from LM 1 to LM 18. |

**
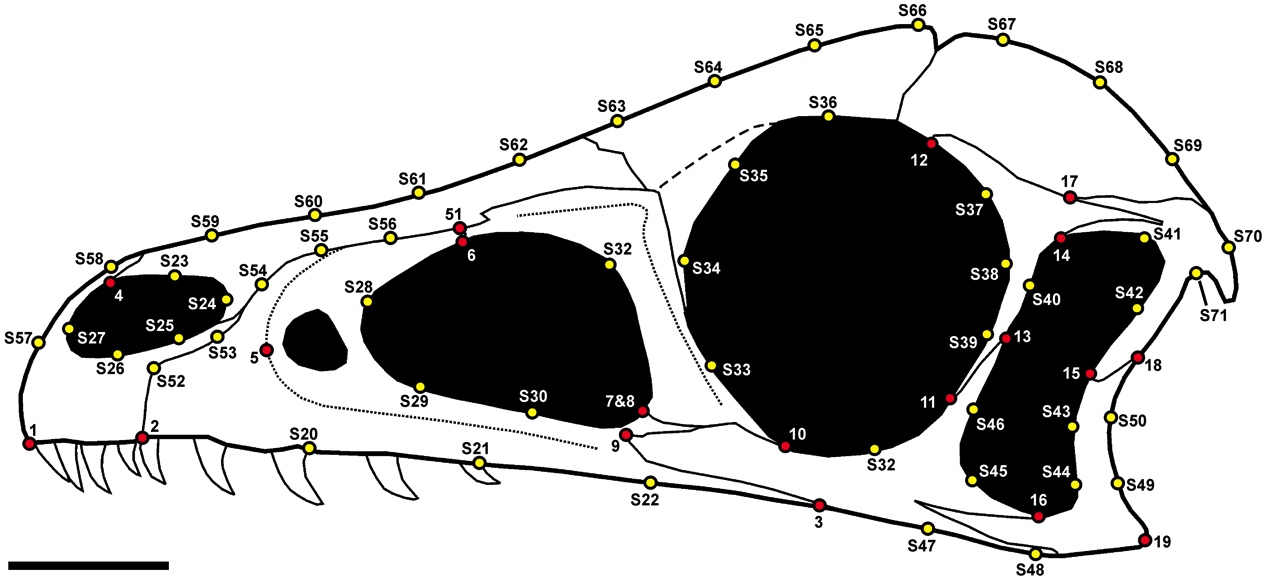
**

**Figure S1** **Illustration of the landmarks and semi-landmarks positions on the skull of *Sciurumimus albersdoerferi*.** Landmarks are shown as red dots, while semi-landmarks are marked with a ‘S’ and are shown as yellow dots. Scale = 1 cm.

**2. Landmark error after Singleton (2002)**

The percent error for digitizing landmarks and semi-landmarks by hand was estimated for the skull reconstruction of the juvenile *Coelophysis* (with n = 10 repetitions) using the method described by Singleton (2002). On the basis of the Procrustes coordinates the mean Procrustes distances to the respective consensus coordinates of each landmark were calculated. Then the relation of these distances to the mean distance of the consensus landmarks to the centroid of the consensus shape was calculated as a percentage of the former from the latter. Landmark and semi-landmark error varies between 0.117 percent (LM 51) and 0.738 percent (LM 3) with a mean of 0.283 percent, having no significant effect on shape analyses.

**Table S2 Percent error for each landmark for the skull of the juvenile specimens of *Coelophysis* with n = 10.**

| **(semi-)LMs** | **%Error** | **(semi-)LMs** | **%Error** | **(semi-)LMs** | **%Error** |
| --- | --- | --- | --- | --- | --- |
| **1** | 0.28511 | **S25** | 0.41778 | **S49** | 0.38979 |
| **2** | 0.25529 | **S26** | 0.34182 | **S50** | 0.73158 |
| **3** | 0.73792 | **S27** | 0.43597 | **51** | 0.11650 |
| **4** | 0.30159 | **S28** | 0.21879 | **S52** | 0.23100 |
| **5** | 0.17860 | **S29** | 0.28964 | **S53** | 0.20120 |
| **6** | 0.26985 | **S30** | 0.24673 | **S54** | 0.17211 |
| **7** | 0.24677 | **S31** | 0.29661 | **S55** | 0.19311 |
| **8** | 0.26012 | **S32** | 0.40849 | **S56** | 0.20648 |
| **9** | 0.16171 | **S33** | 0.26439 | **S57** | 0.20882 |
| **10** | 0.29555 | **S34** | 0.21973 | **S58** | 0.22091 |
| **11** | 0.35026 | **S35** | 0.15236 | **S59** | 0.23261 |
| **12** | 0.26430 | **S36** | 0.16457 | **S60** | 0.16824 |
| **13** | 0.28801 | **S37** | 0.25899 | **S61** | 0.18348 |
| **14** | 0.42348 | **S38** | 0.22989 | **S62** | 0.22272 |
| **15** | 0.19567 | **S39** | 0.26141 | **S63** | 0.21164 |
| **16** | 0.30716 | **S40** | 0.25160 | **S64** | 0.15887 |
| **17** | 0.27963 | **S41** | 0.26707 | **S65** | 0.23221 |
| **18** | 0.24562 | **S42** | 0.25013 | **S66** | 0.20976 |
| **19** | 0.20556 | **S43** | 0.65251 | **S67** | 0.19035 |
| **S20** | 0.27621 | **S44** | 0.24868 | **S68** | 0.20543 |
| **S21** | 0.36408 | **S45** | 0.20666 | **S69** | 0.27989 |
| **S22** | 0.50359 | **S46** | 0.26769 | **S70** | 0.22417 |
| **S23** | 0.32357 | **S47** | 0.52043 | **S71** | 0.27301 |
| **S24** | 0.35938 | **S48** | 0.38044 | **MEAN** | **0.28303** |

**3. Taxon sampling**

**Table S3 List of taxa used in the present analyses with data of occurrences (in million of years, Myr) and sources of images.**

| **Taxa** |  | **Age (Myr)** | **Source** |
| --- | --- | --- | --- |
| *Eoraptor* | Sauropodomorpha | 228.95 | Sereno, Martínez & Alcober, 2013 |
| *Pampadromaeus* | Sauropodomorpha | 228.95 | Cabreira *et al.*, 2011 |
| *Unaysaurus* | Sauropodomorpha | 222.45 | Leal *et al.*, 2004 |
| *Melanorosaurus* | Sauropodomorpha | 214.15 | Yates, 2007 |
| *Plateosaurus* | Sauropodomorpha | 209.65 | Yates, 2003 |
| *Jingshanosaurus* | Sauropodomorpha | 200.30 | Yates, 2012 |
| *Massospondylus* | Sauropodomorpha | 196.05 | See Table S4 |
| *Adeopapposaurus* | Sauropodomorpha | 187.70 | Martínez, 2009 |
| *Shunosaurus* | Sauropodomorpha | 166.90 | Rauhut *et al.*, 2011 |
| *Mamenchisaurus* | Sauropodomorpha | 160.40 | Ouyang & Ye, 2002 |
| *Herrerasaurus* | basal Theropoda | 228.95 | Nesbitt, 2011 |
| *Tawa* | basal Theropoda | 209.50 | Nesbitt, 2011 |
| *Zupaysaurus* | basal Theropoda | 216.00 | modified after Ezcurra, 2007 |
| *Coelophysis* | basal Theropoda | 209.50 | See Table S4 |
| *Syntarsus* | basal Theropoda | 191.00 | Tykoski, 2005 |
| *Limusaurus* | Ceratosauria | 160.40 | Xu *et al*., 2009 |
| *Carnotaurus* | Ceratosauria | 77.85 | Rauhut, 2003 |
| *Majungasaurus* | Ceratosauria | 69.05 | Sampson & Witmer, 2007 |
| Megalosaurid taxon | basal Tetanurae | 166.2 | See Table S4 |
| *Monolophosaurus* | basal Tetanurae | 164.80 | Rauhut, 2003 |
| *Sinraptor* | basal Tetanurae | 160.40 | Currie & Zhao, 1993 |
| *Allosaurus* | basal Tetanurae | 151.15 | See Table S4 |
| *Acrocanthosaurus* | basal Tetanurae | 118.52 | Eddy & Clarke, 2011 |
| Spinosaurid taxon | basal Tetanurae | 127.73 | Rauhut, 2003 |
| *Haplocheirus* | Coelurosauria | 160.40 | own reconstruction |
| *Compsognathus* | Coelurosauria | 148.55 | Peyer, 2006 |
| *Dilong* | Coelurosauria | 126.23 | own reconstruction |
| *Erlikosaurus* | Coelurosauria | 92.05 | Rauhut, 2003 |
| *Garudimimus* | Coelurosauria | 92.05 | Makovicky, Kobayashi & Currie, 2004 |
| *Teratophoneus* | Coelurosauria | 77.05 | Loewen *et al*., 2013 |
| *Gorgosaurus* | Coelurosauria | 77.05 | Carr, 1999 |
| *Daspletosaurus* | Coelurosauria | 77.05 | Rauhut, 2003 |
| *Tarbosaurus* | Coelurosauria | 74.8 | See Table S4 |
| *Tyrannosaurus* | Coelurosauria | 74.75 | Carr & Williamson, 2004 |
| *Alioramus* | Coelurosauria | 69.05 | Brusatte et al., 2009 |

**Table S4 List of ontogenetic trajectories used in the present analyses with data of occurrences (in million of years, Myr) and sources of images.**

| **Taxa** |  | **Age (Myr)** | **Source** |
| --- | --- | --- | --- |
| *Massospondylus* | Sauropodomorpha | 196.05 | Gow, Kitching & Raath, 1990; Reisz *et al.*, 2010 |
| *Coelophysis* | basal Theropoda | 209.50 | Nesbitt, 2011; own reconstruction |
| Megalosaurid taxon | basal Tetanurae | 166.2 | Allain 2002; own reconstruction |
| *Allosaurus* | basal Tetanurae | 151.15 | Loewen, 2009 |
| *Tarbosaurus* | Coelurosauria | 74.8 | Hurum & Sabbath, 2003; modified after Tsuihiji *et al*., 2011 |

**4. Comments on sliding semi-landmarks**

To test the influence of sliding semi-landmarks during general Procrustes analysis (GPA) semi-landmarks were slid in tpsRelW (Rohlf, 2003) to minimize the bending energy and the Procrustes distance, both with a maximal iteration of ten. Afterwards, the resulting Procrustes shapes were compared with that of the regular GPA. The example at hand shows the results for the alignment for the skull of the juvenile *Massospondylus*. The minimization of the bending energy results on artificial elongation of the external naris and the formation of a frontal “crest”-like structure in front of the orbit, while the minimization of the Procrustes distance leads to deformation of the narial shape, the shape of the orbit and the shape of the skull roof in the postorbital region. As these artificial shape alignments affected several taxa within the data set (e.g. *Carnotaurus*, *Compsognathus*, *Mamenchisaurus*, *Shunosaurus*, *Syntarsus*), we decided to treat the semi-landmarks as landmarks. However, to appraise the influence of the semi-landmarks in the original data set, we repeated the shape analyses with a second data set containing only landmark information and compared it with the original results.

**
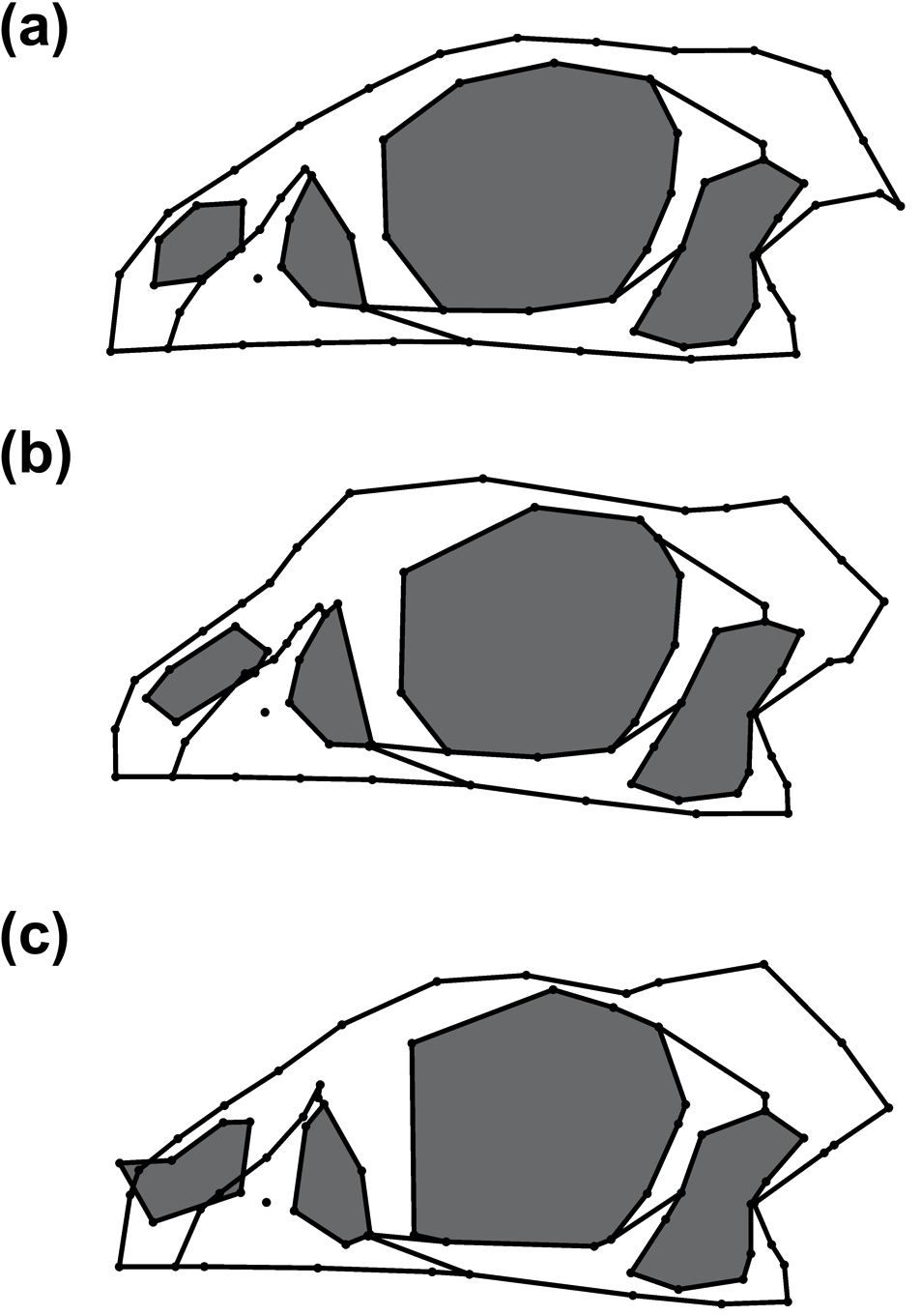
**

**Figure S2 Procrustes shape of the juvenile *Massospondylus* specimen.** (a) Common General Procrustes Analysis (GPA). (b) GPA with slid semi-landmarks minimizing bending energy. (c) GPA with slid semi-landmarks minimizing Procrustes distance. Skull openings are shaded in grey.

**5. Phylogeny**

**
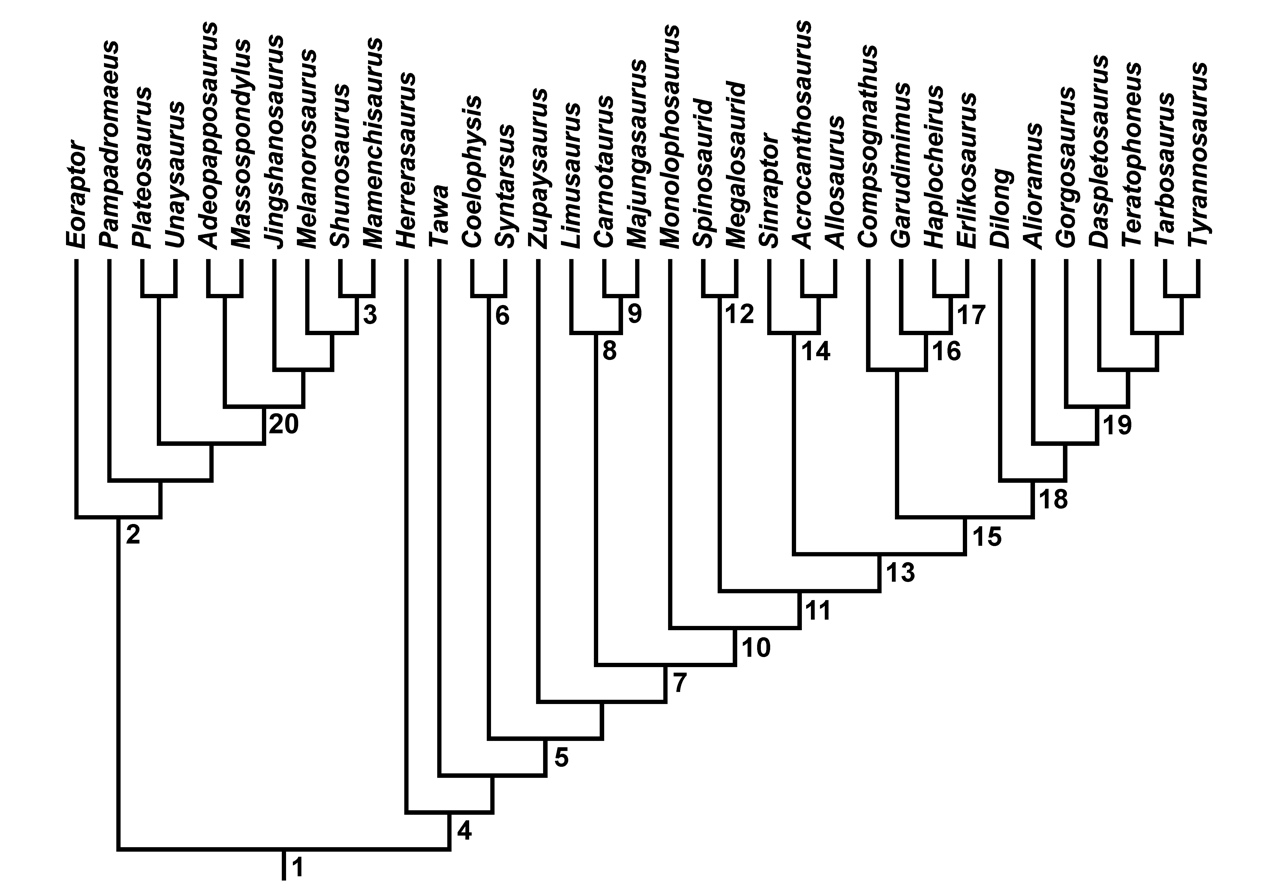
**

**Figure S3 Phylogeny of Saurischia without taxa bearing an enlarged nasal crest. 1** Saurischia, **2** Sauropodomorpha, **3** Eusauropoda, **4** Theropoda, **5** Neotheropoda, **6** Coelophysidae, **7** Averostra, **8** Ceratosauria, **9** Abelisauridae, **10** Tetanurae, **11** Orionides, **12** Megalosauria, **13** Avetheropoda, **14** Allosauroidea, **15** Coelurosauria, **16** Maniraptoriformes, **17** Maniraptora, **18** Tyrannosauroidea, **19** Tyrannosauridae, **20** Massopoda.

**
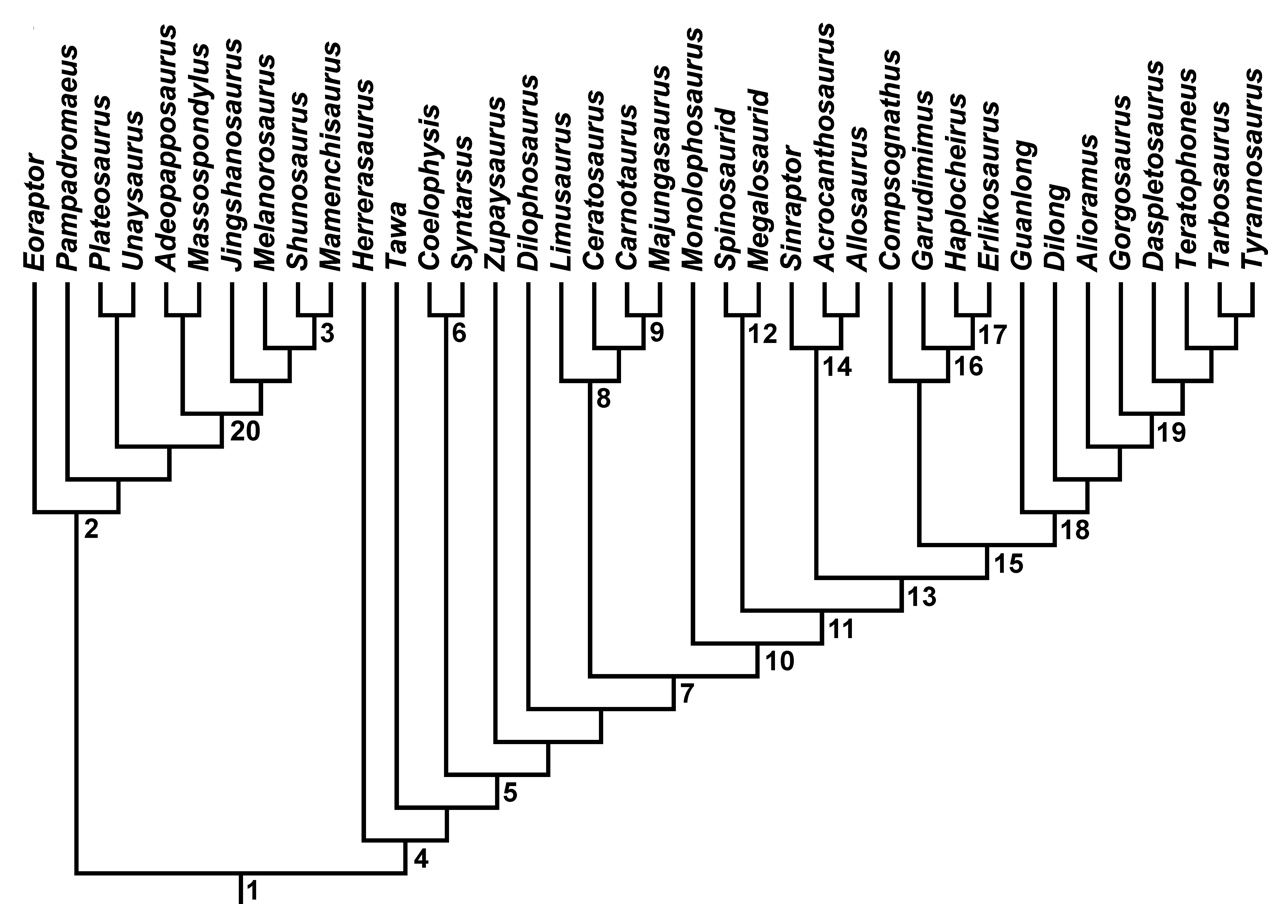
**

**Figure S4 Phylogeny of Saurischia with the crested taxa *Dilophosaurus*, *Ceratosaurus* and *Guanlong* included.**  **1** Saurischia, **2** Sauropodomorpha, **3** Eusauropoda, **4** Theropoda, **5** Neotheropoda, **6** Coelophysidae, **7** Averostra, **8** Ceratosauria, **9** Abelisauridae, **10** Tetanurae, **11** Orionides, **12** Megalosauria, **13** Avetheropoda, **14** Allosauroidea, **15** Coelurosauria, **16** Maniraptoriformes, **17** Maniraptora, **18** Tyrannosauroidea, **19** Tyrannosauridae, **20** Massopoda.

**6. Influence of nasal crests on the results**

**Table S5 List of taxa with nasal crest with data of occurrences (in million of years, Myr) and sources of images.**

| **Taxa** |  | **Age (Myr)** | **Source** |
| --- | --- | --- | --- |
| *Dilophosaurus* | Basal Theropoda | 191.00 | Rauhut, 2003 |
| *Ceratosaurus* | Ceratosauria | 151.15 | Sampson & Witmer, 2007 |
| *Guanlong* | Coelurosauria | 160.40 | Xu *et al.*, 2006 |

To test the influence of taxa with nasal crests, *Dilophosaurus*, *Ceratosaurus* and *Guanlong* were included into the data set (Table S5). After performing GPA the Procrustes coordinates were loaded into Mesquite 2.72 (Maddison & Maddison, 2009) and mapped as continuous characters onto the phylogeny (Fig. S4). For those hypothetical ancestors, which may have been affected by the inclusion of crested taxa (i.e. Averostra, Ceratosauria, Avetheropoda, Coelurosauria and Tyrannosauroidea), the Procrustes coordinates were exported and compared with the respective data of the original data set by computing the percent divergence between both shapes using the method described by Singleton (2002). The inclusion of the crested taxa led to significant shape changes in most of the hypothetical ancestors mentioned above affecting primarily the shape of the skull roof (Table S6, Fig. S5). Here, in the data set containing crested taxa the skull roofs of the hypothetical ancestors show signs of artificial crests, which could potentially falsify the trajectories of the ancestors in the regression analyses, and thus the interpretation of the heterochronic events. Only the shape of hypothetical ancestor of Ceratosauria seems unaffected by the nasal crest of *Ceratosaurus*. Nevertheless, the results of the PCA when crested taxa are included resemble those of original data set (Fig. 2 vs. Fig. S6; Table 1 vs. Table S7; Table S12), indicating that at least trajectories of the terminal taxa are not strongly affected by the inclusion of crested taxa.

**
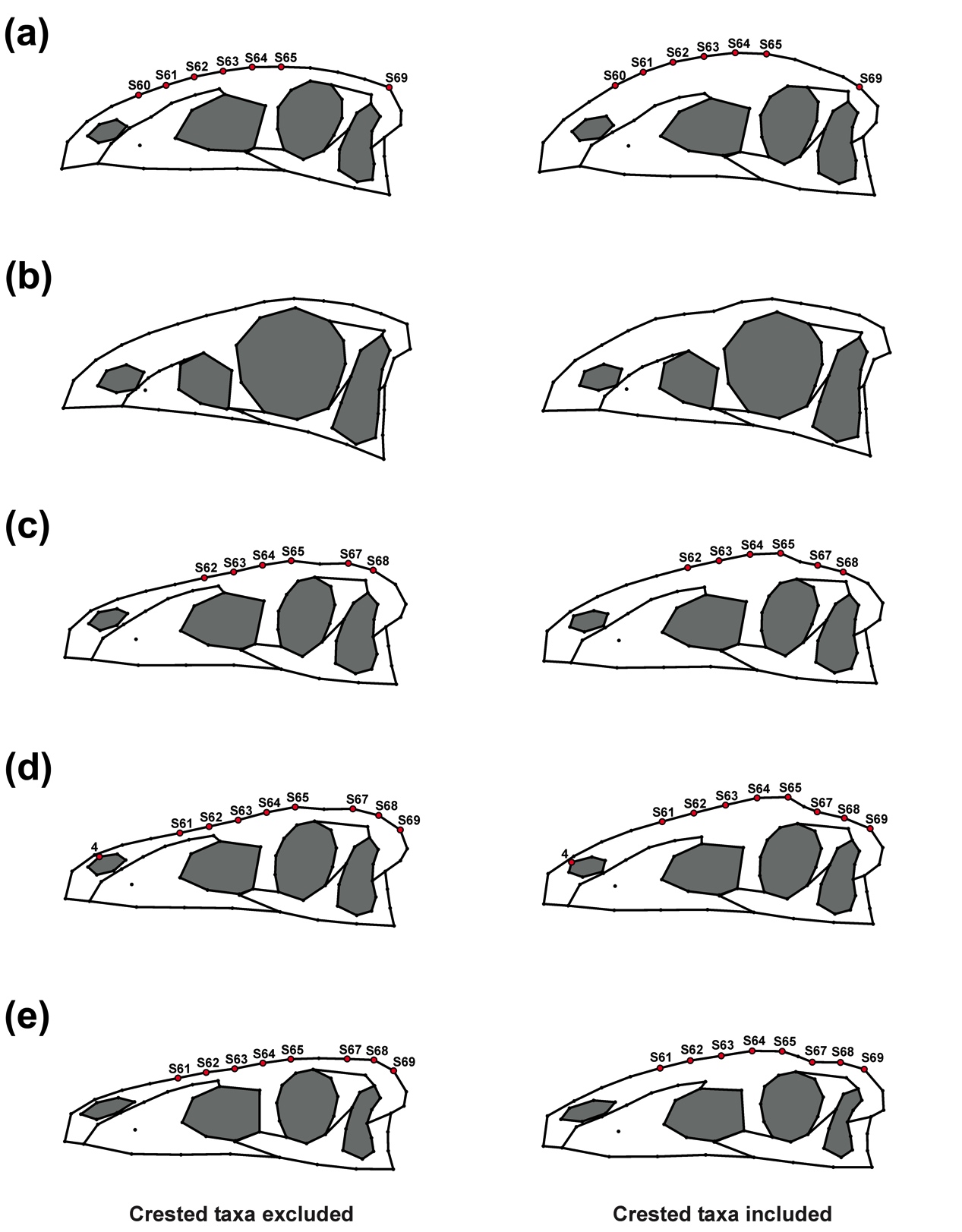
**

**Figure S5 Shape differences in ancestors when crested taxa are included or excluded. (a)** Averostra, **(b)** Ceratosauria, **(c)** Avetheropoda, **(d)** Coelurosauria, **(e)** Tyrannosauroidea (clade including *Dilong* and *Tarbosaurus* with *Guanlong* as outgroup). Red dots mark (semi-)landmarks, which shows significant differences from each other. Skull openings are shaded in grey.

**Table S6 Percentage divergence of Procrustes coordinates of some hypothetical ancestors when crested taxa are included.**

| **LMs** | **Averostra** | **Avetheropoda** | **Coelurosauria** | **Tyrannosauridae** |
| --- | --- | --- | --- | --- |
| **4** | < 5 | < 5 | 5.2137 | < 5 |
| **S60** | 5.2124 | < 5 | < 5 | < 5 |
| **S61** | 6.8402 | < 5 | 5.7688 | 5.0366 |
| **S62** | 7.4140 | 5.2973 | 7.0886 | 6.1963 |
| **S63** | 7.4131 | 6.1263 | 8.2894 | 7.1421 |
| **S64** | 7.3031 | 6.4497 | 8.8181 | 7.5216 |
| **S65** | 6.8977 | 5.7463 | 7.8951 | 6.6629 |
| **S67** | < 5 | 5.8798 | 8.4059 | 8.0697 |
| **S68** | < 5 | 5.5051 | 7.7255 | 7.2441 |
| **S69** | 5.2810 | < 5 | 5.5560 | 5.1951 |

**Table S7 Angles and length of ontogenetic trajectories when crested taxa are included.** Angles of ontogenetic trajectories against PC 1, pairwise angles between ontogenetic trajectories in the PC 1-PC 2, PC 1-PC 3 morphospace and length of ontogenetic trajectories in the PC 1-PC 2 and PC 1-PC 3 morphospace (see Fig. S6). Green fields mark pairwise angles in the PC 1-PC 2 morphospace and orange fields mark that of the PC 1-PC 3 morphospace. Angles, lengths and slopes of ontogenetic trajectories versus log-transformed centroid size (LogCS). See Table 1 for comparison.

|  | ***Massospondylus*** | ***Coelophysis*** | **Megalosaurid taxon** | ***Allosaurus*** | ***Tarbosaurus*** |
| --- | --- | --- | --- | --- | --- |
| **Angle (PC 1-PC 2)** | 86.3695 | 43.1616 | 84.2163 | 5.8852 | 0.1579 |
| **Length (PC 1-PC 2)** | 0.1724 | 0.1176 | 0.1426 | 0.0334 | 0.0406 |
| **Angle (PC 1-PC 3)** | 73.9781 | 7.2269 | 60.9371 | 14.7089 | 59.0058 |
| **Length (PC 1-PC 3)** | 0.0396 | 0.0865 | 0.0296 | 0.0344 | 0.0788 |
|  | ***Massospondylus*** | ***Coelophysis*** | **Megalosaurid taxon** | ***Allosaurus*** | ***Tarbosaurus*** |
| ***Massospondylus*** | 0 | 81.205 | 134.9152 | 91.313 | 165.0277 |
| ***Coelophysis*** | 43.2079 | 0 | 53.7102 | 172.518 | 113.7673 |
| **Megalosaurid taxon** | 2.1533 | 41.0546 | 0 | 133.7718 | 60.0571 |
| ***Allosaurus*** | 87.7453 | 130.9532 | 89.8985 | 0 | 73.7147 |
| ***Tarbosaurus*** | 93.7883 | 136.9962 | 95.9416 | 6.0431 | 0 |
|  | ***Massospondylus*** | ***Coelophysis*** | **Megalosaurid taxon** | ***Allosaurus*** | ***Tarbosaurus*** |
| **Angle (LogCS)** | 3.3623 | 4.8561 | 4.6047 | 0.2805 | 1.5869 |
| **Length (LogCS)** | 2.2815 | 1.0636 | 1.8147 | 1.0657 | 1.4016 |
| **Slope (LogCS)** | 0.0588 | 0.0850 | 0.0805 | 0.0049 | 0.0277 |

**
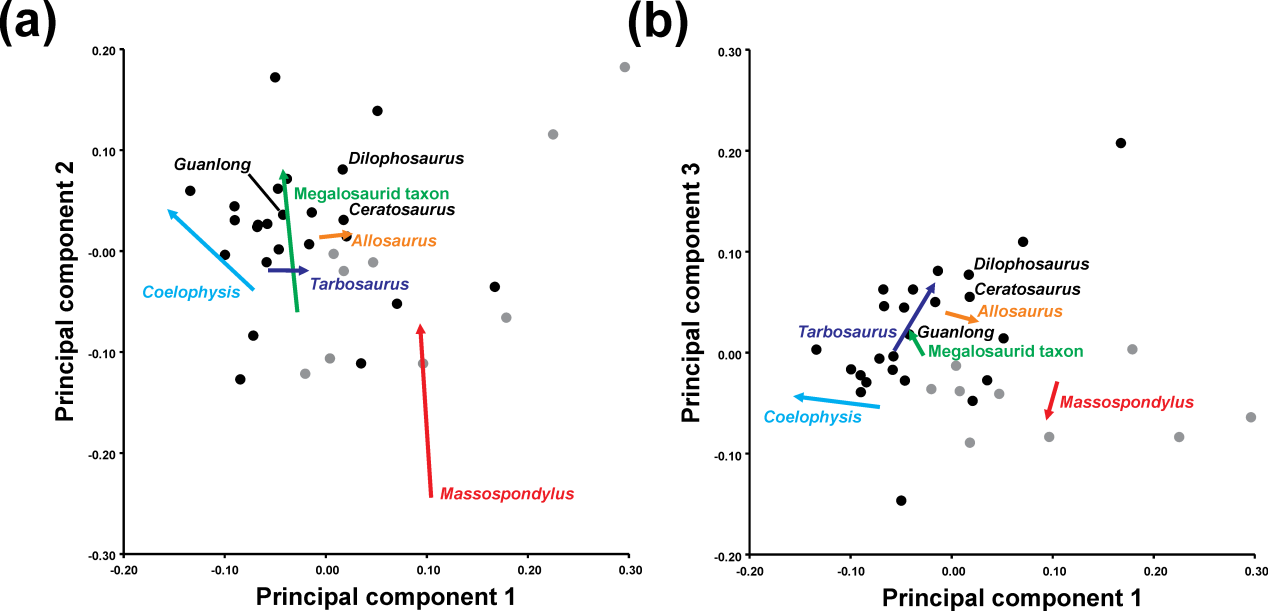
**

**Figure S6 Principal component analysis when crested taxa are included.** (a) Ontogenetic trajectories of terminal taxa for PC 1 against PC 2. (b) Ontogenetic trajectories of terminal taxa for PC 1 against PC 3. Theropod taxa are shown as black dots, while sauropodomorph taxa are shown as grey dots. The arrows illustrate the different ontogenetic trajectories, in which the arrowhead marks the position of the adult individual. When compared to the original data of the main analysis the ontogenetic trajectories of the terminal taxa do not change when crested theropods are included in the data set (see Table 1 vs. Table S7; Table S12, Fig. 2).

**7. Influence of semi-landmarks on the results**

Exclusion of semi-landmarks from the data set led to five differently directed ontogenetic trajectories when a PCA is performed (PC 1: 28.4 %; PC 2: 20.0 %; PC 3: 15.6 %). The ontogenetic trajectory of the megalosaurid taxon is explained approximately equally by the first three PCs, that of *Allosaurus* is mainly captured by PC 1 and 2, and that of *Tarbosaurus* is explained by PCs 1 and 3. As in the main data set the ontogenetic trajectories of *Allosaurus* and *Tarbosaurus* are rather short compared to the other species. The ontogenetic trajectories of *Massospondylus* and *Coelophysis* are mainly explained by shape variation captured by PCs 2 and 3, in which the ontogenetic trajectory of *Massospondylus* along PC 3 is directed opposite to that of *Coelophysis*, *Tarbosaurus* and the megalosaurid taxon (see Fig. S7a, b; Table S8). However, based on the length of the trajectories most of the ontogenetic variation in *Massospondylus* and *Coelophysis* is captured by the second principal component axis.

Comparing only the terminal and ancestral ontogenetic trajectories with each other in a PCA (PC 1: 41.7 %; PC 2: 28.3 %) the ontogenetic trajectories of *Massospondylus*, *Coelophysis*, and the hypothetical ancestors of Neotheropoda and Avetheropoda are mainly influenced by the first PC axis, in which the trajectories of *Massospondylus* and *Coelophysis* have a negative slope with respect to PC2, while the slope of Neotheropoda and Avetheropoda is positive. In the megalosaurid taxon*, Allosaurus*, and the hypothetical ancestors of Saurischia and Orionides the ontogenetic variation is influenced by PC 1 and PC 2, while that of *Tarbosaurus* is mainly influenced by PC 2 (see Fig. S7c; Table S9).

When semi-landmarks are excluded from the data set, most of the heterochronic trends found in the regression analysis are similar to those described in the original analysis (see Fig. S8 vs. Fig. 4; Table S11 vs. Table 4). The results differ from the original data set as most changes were found to be not significant. Only for the megalosaurid taxon and the hypothetical ancestor of Neotheropoda a possible cranial peramorphosis could be detected. Thus, most of the significant shape changes found in the main sample took place in skull areas captured by semi-landmarks, which include the skull outline, but also the relative size and shape of the skull openings.

**Table S8 Angles between terminal ontogenetic trajectories when semi-landmarks are excluded.** Angles of ontogenetic trajectories against PC 1, pairwise angles between ontogenetic trajectories in the PC 1-PC 2 and PC 1-PC 3 morphospace and length of ontogenetic trajectories in the PC 1-PC 2 and PC 1-PC 3 morphospace (Fig. S7a, b). Green fields mark pairwise angles in the PC 1-PC 2 morphospace and orange fields mark that of the PC 1-PC 3 morphospace. Angles, lengths and slopes of ontogenetic trajectories versus log-transformed centroid size (LogCS) (Fig. S8a).

|  | ***Massospondylus*** | ***Coelophysis*** | **Megalosaurid taxon** | ***Allosaurus*** | ***Tarbosaurus*** |
| --- | --- | --- | --- | --- | --- |
| **Angle (PC 1-PC 2)** | 81.171 | 81.3625 | 52.1091 | 32.4126 | 1.1564 |
| **Length (PC 1-PC 2)** | 0.1346 | 0.1555 | 0.1428 | 0.0575 | 0.0409 |
| **Angle (PC 1-PC 3)** | 59.6961 | 32.3194 | 23.0962 | 7.4444 | 55.0223 |
| **Length (PC 1-PC 3)** | 0.0409 | 0.0276 | 0.0954 | 0.049 | 0.0713 |
|  | ***Massospondylus*** | ***Coelophysis*** | **Megalosaurid taxon** | ***Allosaurus*** | ***Tarbosaurus*** |
| ***Massospondylus*** | 0 | 152.6233 | 82.7922 | 52.2517 | 114.7184 |
| ***Coelophysis*** | 17.4665 | 0 | 124.5844 | 155.125 | 92.6583 |
| **Megalosaurid taxon** | 29.062 | 46.5285 | 0 | 30.5406 | 31.9262 |
| ***Allosaurus*** | 48.7585 | 66.2249 | 19.6965 | 0 | 62.4667 |
| ***Tarbosaurus*** | 80.0146 | 97.4811 | 50.9526 | 31.2562 | 0 |
|  | ***Massospondylus*** | ***Coelophysis*** | **Megalosaurid taxon** | ***Allosaurus*** | ***Tarbosaurus*** |
| **Angle (LogCS)** | 2.5478 | 6.7957 | 4.3554 | 2.3132 | 1.8715 |
| **Length (LogCS)** | 2.295 | 1.0726 | 1.8354 | 1.0259 | 1.4104 |
| **Slope (logCS)** | 0.0445 | 0.1192 | 0.0762 | 0.0404 | 0.0327 |

**Table S9 Angles between terminal and ancestral ontogenetic trajectories when semi-landmarks are excluded.** Angles of ontogenetic trajectories against PC 1, pairwise angles between ontogenetic trajectories in the PC 1-PC 2 morphospace and length of ontogenetic trajectories in the PC 1-PC 2 morphospace (Fig. S7c).

|  | **Saurischia** | ***Massospondylus*** | **Neotheropoda** | ***Coelophysis*** | **Orionides** | **Megalosaurid taxon** | **Avetheropoda** | ***Allosaurus*** | ***Tarbosaurus*** |
| --- | --- | --- | --- | --- | --- | --- | --- | --- | --- |
| **Angle (PC 1-PC 2)** | 35.2317 | 7.4692 | 14.7513 | 7.7407 | 21.9848 | 37.8587 | 8.2562 | 58.0965 | 76.9355 |
| **Length (PC 1-PC 2)** | 0.0817 | 0.124 | 0.1257 | 0.1597 | 0.0949 | 0.1464 | 0.0625 | 0.0487 | 0.0722 |
|  | **Saurischia** | ***Massospondylus*** | **Neotheropoda** | ***Coelophysis*** | **Orionides** | **Megalosaurid taxon** | **Avetheropoda** | ***Allosaurus*** | ***Tarbosaurus*** |
| **Saurischia** | 0 |  |  |  |  |  |  |  |  |
| ***Massospondylus*** | 42.7009 | 0 |  |  |  |  |  |  |  |
| **Neotheropoda** | 20.4805 | 22.2204 | 0 |  |  |  |  |  |  |
| ***Coelophysis*** | 42.9724 | 0.2715 | 22.4919 | 0 |  |  |  |  |  |
| **Orionides** | 13.247 | 29.4539 | 7.2335 | 29.7255 | 0 |  |  |  |  |
| **Megalosaurid taxon** | 2.627 | 45.3279 | 23.1075 | 45.5994 | 15.874 | 0 |  |  |  |
| **Avetheropoda** | 26.9755 | 15.7254 | 6.495 | 15.9969 | 13.7286 | 29.6025 | 0 |  |  |
| ***Allosaurus*** | 22.8648 | 65.5657 | 43.3453 | 65.8372 | 36.1118 | 20.2378 | 49.8403 | 0 |  |
| ***Tarbosaurus*** | 41.7038 | 84.4047 | 62.1843 | 84.6762 | 54.9508 | 39.0768 | 68.6793 | 18.839 | 0 |

**Table S10 Angles between terminal and ancestral ontogenetic trajectories when semi-landmarks are excluded.** Angles, lengths and slopes of ontogenetic trajectories from the regression of shape (Regression score, RS and Euclidean Distance, ED) versus log-transformed centroid size (LogCS) (Fig. 8b, c).

| **Regression (RS)** | **Saurischia** | ***Massospondylus*** | **Neotheropoda** | ***Coelophysis*** | **Orionides** | **Megalosaurid taxon** | **Avetheropoda** | ***Allosaurus*** | ***Tarbosaurus*** |
| --- | --- | --- | --- | --- | --- | --- | --- | --- | --- |
| **Angle (LogCS)** | 4.2406 | 3.4176 | 5.2886 | 7.5316 | 4.3657 | 4.332 | 3.8961 | 3.1168 | 1.5063 |
| **Length (LogCS)** | 1.1018 | 2.2968 | 1.3944 | 1.0744 | 1.3255 | 1.8353 | 0.9593 | 1.0266 | 1.4101 |
| **Slope (LogCS)** | 0.0741 | 0.0597 | 0.0926 | 0.1322 | 0.0763 | 0.0758 | 0.0681 | 0.0545 | 0.0263 |
| **Regression (ED)** | **Saurischia** | ***Massospondylus*** | **Neotheropoda** | ***Coelophysis*** | **Orionides** | **Megalosaurid taxon** | **Avetheropoda** | ***Allosaurus*** | ***Tarbosaurus*** |
| **Angle (LogCS)** | 1.7660 | 4.7098 | 3.2732 | 6.2267 | 2.7699 | 2.2483 | 2.9149 | 1.8653 | 0.2418 |
| **Length (LogCS)** | 1.0993 | 2.3005 | 1.3908 | 1.0714 | 1.3232 | 1.8315 | 0.9583 | 1.0257 | 1.4096 |
| **Slope (LogCS)** | 0.0308 | 0.0824 | 0.0572 | 0.1091 | 0.0484 | 0.0393 | 0.0509 | 0.0326 | 0.0042 |

**
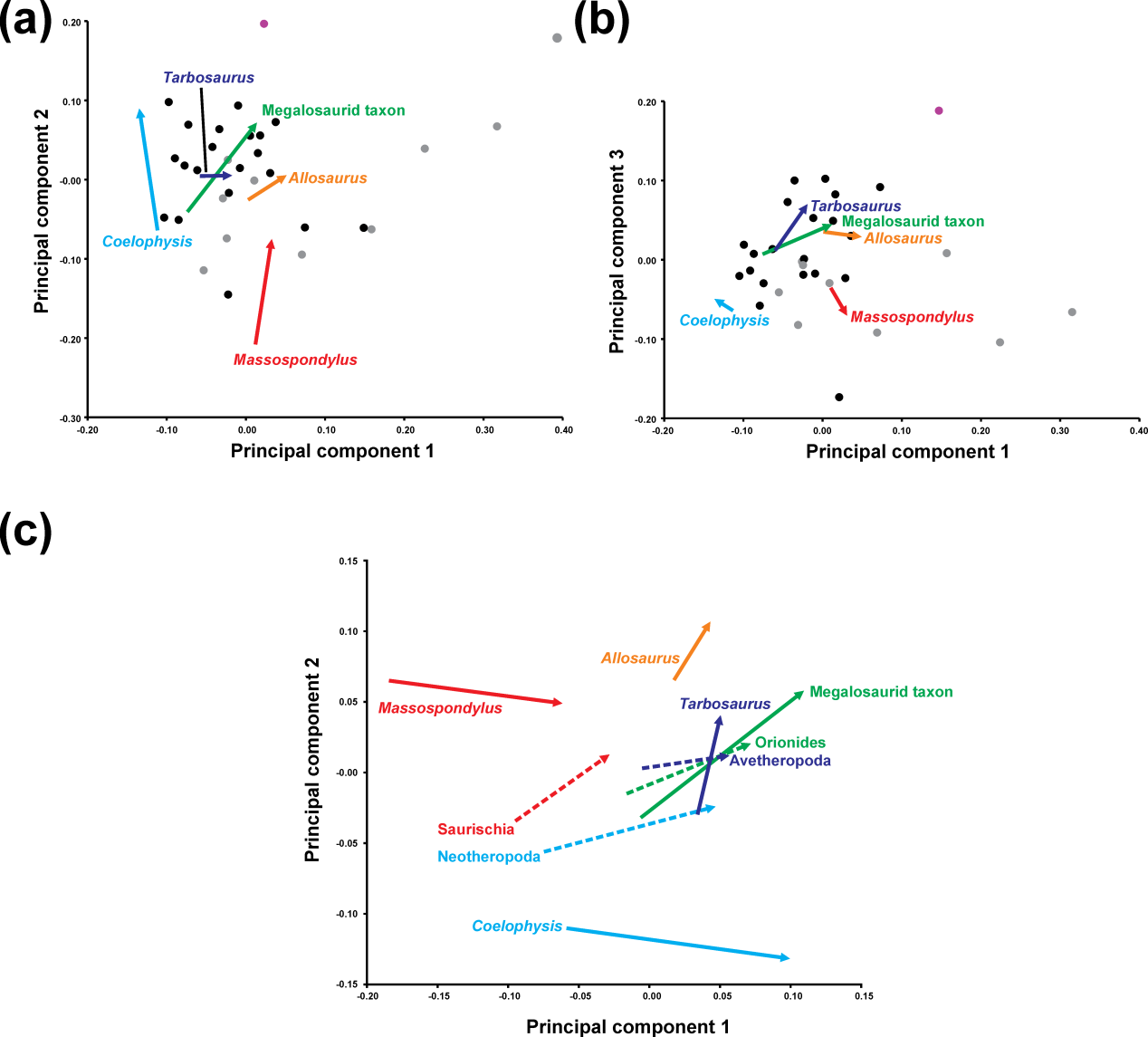
**

**Figure S7 Principal component analysis when semi-landmarks are excluded.** (a) Ontogenetic trajectories of terminal taxa for PC 1 versus PC 2. (b) Ontogenetic trajectories of terminal taxa for PC 1 against PC 3. Theropod taxa are shown as black dots, while sauropodomorph taxa are shown as grey dots. (c) Terminal and ancestral ontogenetic trajectories for PC 1 against PC 2. The arrows illustrate the different ontogenetic trajectories, in which the arrowhead marks the position of the adult individual.

**
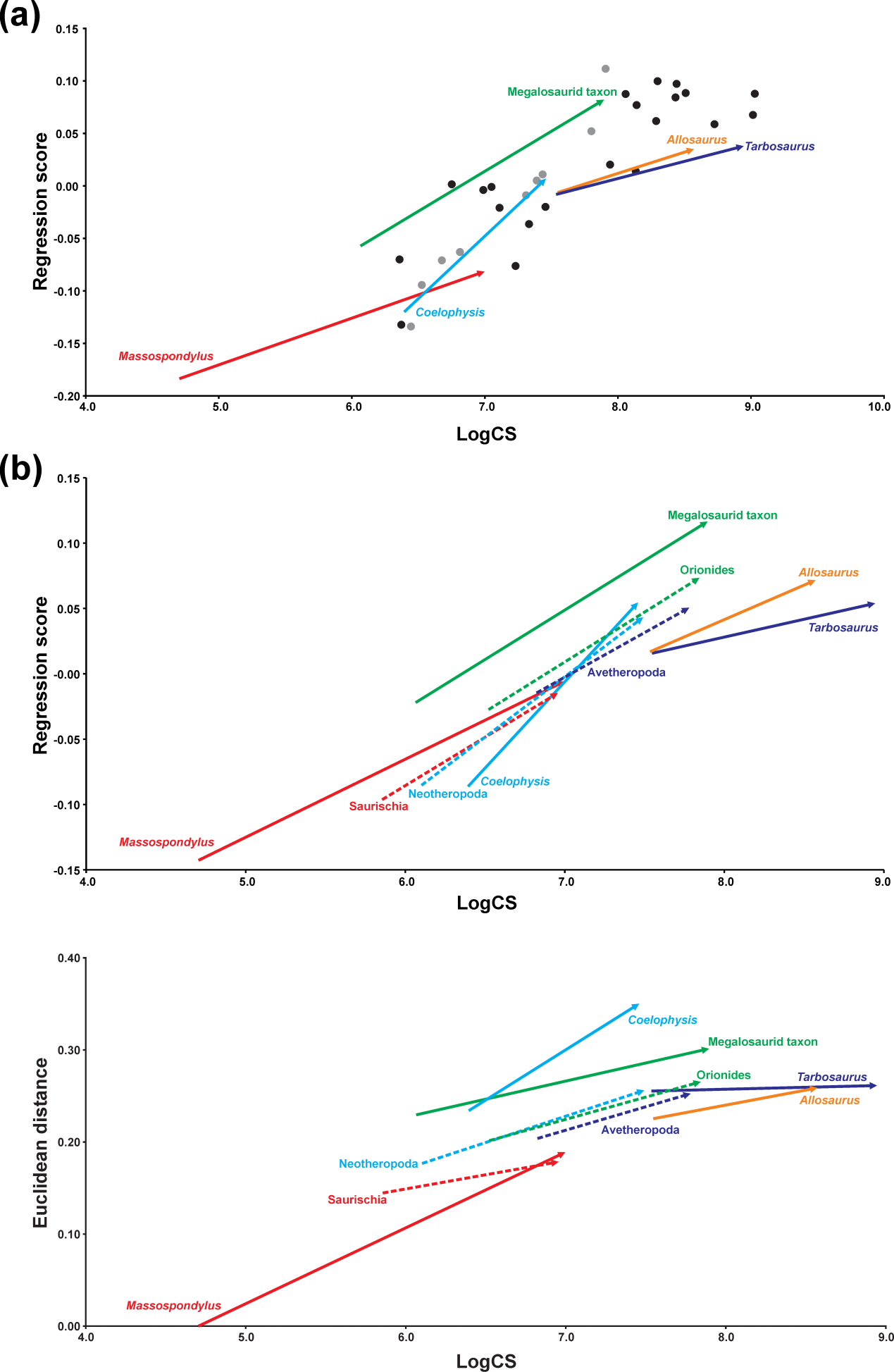
**

**Figure S8 Regression analysis when semi-landmarks are excluded.** (a) Regression analysis of all terminal taxa including ontogenetic trajectories against log-transformed skull centroid size (LogCS) (p < 0.0001). (b) Regression analysis of only terminal (solid arrows) and ancestral (dashed arrows) ontogenetic trajectories against log-transformed centroid size (p < 0.0001). Theropod taxa are shown as black dots, while sauropodomorph taxa are shown as grey dots. The arrows illustrate the different ontogenetic trajectories, in which the arrowhead marks the position of the adult individual.

**Table S11 Overview of heterochronies in saurischian skull shape when semi-landmarks are excluded.** The table shows the differences of the regression scores (ΔRS) and the Euclidean distances (ΔED) between ancestor-descendent relationships of adult individuals from the regression analysis (Fig. S8b, c) and the interpretation regarding heterochrony. ΔRS and ΔED values in brackets mark non-significant trends. NA = not available.

|  | **ΔRS** | **ΔED** | **Heterochrony** |
| --- | --- | --- | --- |
| **Saurischia-*Massospondylus*** | (0.0088) | (0.0104) | NA |
| **Saurischia-Neotheropoda** | 0.0579 | 0.0776 | Peramorphosis |
| **Neotheropoda-*Coelophysis*** | (0.0114) | 0.0941 | NA |
| **Neotheropoda-Orionides** | 0.0303 | (0.0093) | NA |
| **Orionides-megalosaurid taxon** | 0.0430 | 0.0359 | Peramorphosis |
| **Orionides-Avetheropoda** | -0.0229 | (-0.0129) | NA |
| **Avetheropoda-*Allosaurus*** | 0.0210 | (0.0062) | NA |
| **Avetheropoda-*Tarbosaurus*** | (0.0033) | (0.0088) | NA |
| **95 % CIs** | 0.0079 | 0.0095 |  |
| **Significance levels (p=0.05)** | 0.0119 | 0.0143 |  |

**Table S12 Comparison of the results of the PCA and the regression test (shape vs. log-transformed centroid size) for all tree data sets.**

|  | **PCA** | | | **Regression** | |
| --- | --- | --- | --- | --- | --- |
|  | **PC 1** | **PC 2** | **PC 3** | **Correlation** | **p value** |
| **All Saurischia (original)** | 30.75% | 23.94% | 13.26% | 15.32% | <0.0001 |
| **All Saurischia (original) – PC 1** |  |  |  | 0.46% | 0.6749 |
| **All Saurischia (original) – PC 2** |  |  |  | 45.68% | <0.0001 |
| **All Saurischia (original) – PC 3** |  |  |  | 27.30% | 0.0004 |
| **All Saurischia (including crested taxa)** | 28.58% | 22.73% | 12.96% | 14.71% | <0.0001 |
| **All Saurischia (without semi- landmarks)** | 28.37% | 19.98% | 15.64% | 14.98% | <0.0001 |
| **Only Ontogenetic trajectories (original)** | 50.39% | 20.79% | 8.17% | 48.19% | <0.0001 |
| **Only Ontogenetic trajectories (original) – PC 1** |  |  |  | 51.35% | 0.0011 |
| **Only Ontogenetic trajectories (original) – PC 2** |  |  |  | 30.28% | 0.0180 |
| **Only Ontogenetic trajectories (without semi- landmarks)** | 41.74% | 28.27% | 8.27% | 50.84% | <0.0001 |

**8. References**

Allain R. 2002. Discovery of megalosaur (Dinosauria, Theropoda) in the middle Bathonian of Normandy (France) and its implications for the phylogeny of basal Tetanurae. *Journal of Vertebrate Paleontology* 22:548–563.

Brusatte SL, Carr TD, Erickson GM, Bever GS, Norell MA. 2009. A long-snouted, multihorned tyrannosaurid from the Late Cretaceous of Mongolia. *Proceedings of the National Academy of Sciences* 106:17261–17266.

Cabreira SF, Schultz CL, Bittencourt JS, Soares MB, Fortier DC, Silva LR, Langer MC. 2011. New stem-sauropodomorph (Dinosauria, Saurischia) from the Triassic of Brazil. *Naturwissenschaften* 98:1035–1040.

Carr TD. 1999. Craniofacial ontogeny in Tyrannosauridae (Dinosauria, Coelurosauria). *Journal of Vertebrate Paleontology* 19:497–520.

Carr TD, Williamson TE. 2004. Diversity of late Maastrichtian Tyrannosauridae (Dinosauria: Theropoda) from western North America. *Zoological Journal of the Linnean Society* 142:479–523.

Currie PJ, Zhao X. 1993. A new carnosaur (Dinosauria, Theropoda) from the Jurassic of Xinjiang, People’s Republic of China. *Canadian Journal of Earth Sciences* 30:2037–2081.

Eddy DR, Clarke JA. 2011. New information on the cranial anatomy of *Acrocanthosaurus atokensis* and its implications for the phylogeny of Allosauroidea (Dinosauria: Theropoda). *PLoS ONE* 6:e17932.

Ezcurra MD. 2007. The cranial anatomy of the coelophysoid theropod *Zupaysaurus rougieri* from the Upper Triassic of Argentina. *Historical Biology* 19:185–202.

Gow CE, Kitching JW, Raath MA. 1990. Skulls of the prosauropod dinosaur *Massospondylus carinatus* Owen in the collections of the Bernand Price Institute for Palaeontological Research. *Palaeontologia Africana* 27:45–58.

Hurum JH, Sabath K. 2003. Giant theropod dinosaurs from Asia and North America: skulls of *Tarbosaurus bataar* and *Tyrannosaurus rex* compared. *Acta Palaeontologica Polonica* 48:161–190.

Leal LA, Azevedo SAK, Kellner AWA, Da Rosa AAS. 2004. A new early dinosaur (Sauropodomorpha) from the Caturrita Formation (Late Triassic), Paraná Basin, Brazil. *Zootaxa* 690:1–24.

Loewen MA. 2009. *Variation in the Late Jurassic theropod dinosaur* Allosaurus*: ontogenetic, functional, and taxonomic implications*. University of Utah, Salt Lake City.

Loewen MA, Irmis RB, Sertich JJW, Currie PJ, Sampson SD. 2013. Tyrant dinosaur evolution tracks the rise and fall of Late Cretaceous oceans. *PLoS ONE* 8:e79420.

Maddison WP, Maddison DR. 2009. Mesquite: A modular system of evolutionary analysis. Version 2.72.

Makovicky PJ, Kobayashi Y, Currie PJ. 2004. Ornithimosauria. In: Weishampel DB, Dodson P, Osmólska H eds. *The Dinosauria*. Berkeley: University of California Press, 137–150.

Martínez RN. 2009. *Adeopapposaurus mognai*, gen. et sp. nov. (Dinosauria: Sauropodomorpha), with comments on adaptations of basal Sauropodomorpha. *Journal of Vertebrate Paleontology* 29:142–164.

Nesbitt SJ. 2011. The early evolution of archosaurs: relationships and the origin of major clades. *Bulletin of the American Museum of Natural History* 352:1–292.

Ouyang H, Ye Y. 2002. *The first mamenchisaurian skeleton with complete skull,* Mamenchisaurus youngi. Chengdu: Sichuan Science and Technology Press.

Peyer K. 2006. A reconsideration of *Compsognathus* from the Upper Tithonian of Canjuers, southeastern France. *Journal of Vertebrate Paleontology* 26:879–896.

Rauhut OWM. 2003. The interrelationships and evolution of basal theropod dinosaurs. *Special Papers in Palaeontology* 69:1–213.

Rauhut OWM, Fechner R, Remes K, Reis K. 2011. How to get big in the Mesozoic: the evolution of the sauropodomorph body plan. In: Klein N, Remes K, Gee CT, Sander PM eds. *Biology of the sauropod dinosaurs: understanding the life of giants*. Bloomington: Indiana University Press, 119–149.

Reisz RR, Evans DC, Sues H-D, Scott D. 2010. Embryonic skeletal anatomy of the sauropodomorph dinosaur *Massospondylus* from the Lower Jurassic of South Africa. *Journal of Vertebrate Paleontology* 30:1653–1665.

Rohlf FJ. 2003. tpsRelw, relative warps analysis, version 1.36.

Sampson SD, Witmer LM. 2007. Craniofacial anatomy of *Majungasaurus crenatissimus* (Theropoda: Abelisauridae) from the Late Cretaceous of Madagascar. *Society of Vertebrate Paleontology Memoir* 8:32–102.

Sereno PC, Martínez RN, Alcober OA. 2013. Osteology of *Eoraptor lunensis* (Dinosauria, Sauropodomorpha). *Society of Vertebrate Paleontology Memoir* 12:83–179.

Singleton M. 2002. Patterns of cranial shape variation in the *Papionini* (Primates: Cercopithecinae). *Journal of Human Evolution* 42:547–578.

Tsuihiji T, Watabe M, Tsogtbaatar K, Tsubamoto T, Barsbold R, Suzuki S, Lee AH, Ridgely RC, Kawahara Y, Witmer LM. 2011. Cranial osteology of a juvenile specimens of *Tarbosaurus bataar* (Theropoda, Tyrannosauridae) from the Nemegt Formation (Upper Cretaceous) of Bugin Tsav, Mongolia. *Journal of Vertebrate Paleontology* 31:497–517.

Tykoski RS. 2005. *Anatomy, ontogeny, and phylogeny of Coelophysoid theropods*. The University of Texas, Austin.

Xu X, Clark JM, Forster CA, Norell MA, Erickson GM, Eberth DA, Jia C, Zhao Q. 2006. A basal tyrannosauroid dinosaur from the Late Jurassic of China. *Nature* 439:715–718.

Xu L, Clark JM, Mo J, Choiniere JN, Forster CA, Erickson GM, Hone DWE, Sullivan C, Eberth DA, Nesbitt SJ, Zhao Q, Hernandez R, Jia C, Han F, Guo Y. 2009. A Jurassic ceratosaur from China helps clarify avian digital homologies. *Nature* 459:940–944.

Yates AM. 2003. The species taxonomy of the sauropodomorph dinosaurs from the Löwenstein Formation (Norian, Late Triassic) of Germany. *Palaeontology* 46:317–337.

Yates AM. 2007. The first complete skull of the Triassic dinosaur *Melanorosaurus* Haughton (Sauropodomorpha: Anchisauria). *Special Papers in Palaeontology* 77:9–55.

Yates AM. 2012. Basal Sauropodomorpha: the “prosauropods.” In: Brett-Surman MK, Holtz TRJ, Farlow JO eds. *The complete dinosaur*. Bloomington: Indiana University Press, 425–444.
